# Gender Differences in Depressive and Anxiety Symptoms During the First Stage of the COVID-19 Pandemic: A Cross-Sectional Study in Latin America and the Caribbean

**DOI:** 10.3389/fpsyt.2022.727034

**Published:** 2022-03-17

**Authors:** Percy Herrera-Añazco, Diego Urrunaga-Pastor, Vicente A. Benites-Zapata, Guido Bendezu-Quispe, Carlos J. Toro-Huamanchumo, Adrian V. Hernandez

**Affiliations:** ^1^Universidad Privada San Juan Bautista, Escuela de enfermería, Lima, Peru; ^2^Red Internacional en Salud Colectiva y Salud Intercultural, Mexico, Mexico; ^3^Instituto de Evaluación de Tecnologías en Salud e Investigación–IETSI, Dirección de Investigación en Salud, EsSalud, Lima, Peru; ^4^Universidad Científica del Sur, Facultad de Ciencias de la Salud, Lima, Peru; ^5^Universidad San Ignacio de Loyola, Unidad para la Generación y Síntesis de Evidencias en Salud, Lima, Peru; ^6^Universidad Privada Norbert Wiener, Centro de Investigación Epidemiológica en Salud Global, Lima, Peru; ^7^Clínica Avendaño, Unidad de Investigación Multidisciplinaria, Lima, Peru; ^8^Health Outcomes, Policy and Evidence Synthesis (HOPES) Group, University of Connecticut School of Pharmacy, Storrs, CT, United States; ^9^Universidad San Ignacio de Loyola, Unidad de Revisiones Sistemáticas y Metaanálisis, Guías de Práctica Clínica y Evaluaciones Tecnológicas Sanitarias, Lima, Peru

**Keywords:** Latin America, anxiety, depression, gender identity, COVID-19

## Abstract

**Background:**

Previous studies have suggested that the pandemic impact on mental health could vary according to gender. We aimed to evaluate the gender influence in the prevalence of depressive and anxiety symptoms in Latin American and the Caribbean (LAC) countries in the first stage of the COVID-19 pandemic.

**Methods:**

We conducted a secondary analysis employing the Facebook–COVID-19 Symptom Survey developed by the University of Maryland. We categorized gender as men, women, and non-binary. The outcomes were the presence of anxiety or depressive symptoms, measured with two adapted questions extracted from the Kessler Psychological Distress Scale (K10). We used generalized linear models from the Poisson family, considering the survey's complex sampling. We calculated crude and adjusted prevalence ratios (PR) with their 95% confidence intervals (95% CI) and explored interactions with gender using the adjusted Wald test.

**Results:**

We included 1,338,320 adults from LAC countries; 48.0, 50.6, and 1.4% were men, women, and non-binary participants, respectively. The overall prevalence of anxiety or depressive symptoms was 44.8 and 46.6%, respectively. We found interactions between gender and the rest of the independent variables. In the non-binary group, the association between age and anxiety symptomatology was lost after an age of 55 years. Furthermore, whereas living in a town was associated with a lower prevalence of anxiety and depression symptomatology in men and women, this did not happen among non-binary individuals. Compliance with physical distancing was associated with a lower prevalence of anxiety and depression symptomatology among women (anxiety: PRa = 0.98; 95% CI = 0.97–0.99; *p* < 0.001, depression: PRa = 0.96; 95% CI = 0.95–0.97; *p* < 0.001) and only anxiety in non-binary participants (anxiety: PRa = 0.92; 95% CI = 0.88–0.98; *p* = 0.005). This was not evidenced among men participants (anxiety: PRa = 0.99; 95% CI = 0.96–1.01; *p* = 0.199, depression: PRa = 0.98; 95% CI = 0.96–1.00; *p* = 0.084). In addition, compliance with handwashing was associated with a higher prevalence of anxiety symptomatology among men (PRa = 1.06; 95% CI = 1.05–1.11; *p* < 0.001) and women participants (PRa = 1.03; 95% CI = 1.01–1.05; *p* = 0.016).

**Conclusion:**

Approximately 4 out of 10 participants had anxiety or depressive symptoms. Women and non-binary gender people had more symptoms of anxiety or depression. The factors associated with these symptoms varied according to gender. It is essential to evaluate gender-related strategies to improve mental health during the COVID-19 pandemic.

## Introduction

The COVID-19 pandemic produced substantial adjustments worldwide in people's lifestyles and catastrophic costs to nation's economies (1, 2). To lessen the impact of the pandemic, national quarantines were initiated in the first months of the crisis in the Latin American and the Caribbean (LAC) countries, in order to expand the care capacity of the health systems of these nations, which are characterized by problems of governance and infrastructure, and a lack of qualified human resources limiting patient care during the peak of the pandemic (3–6).

Although not present in LAC, in recent decades, there have been outbreaks of viral infections that have required quarantines in some Asian countries, which increased the presence of factors associated with stress and anxiety described during these periods of quarantine (7–11). These factors include the risk of being infected, death and infection of relatives and friends, feeling of loneliness and social isolation, physical and emotional fatigue of health workers, massive loss of employment, financial insecurity and poverty, and infodemic (12–14). Previous studies involving infected patients and people in quarantine found significant levels of psychological distress, hopelessness, anxiety, depression, anger, and fear of contagion (7–11). Likewise, a meta-analysis involving 25 studies developed in the course of severe acute respiratory syndrome (SARS) and Middle Eastern respiratory syndrome (MERS) outbreaks, both part of the coronavirus family, described that patients experienced significant effects such as anxiety and lack of sleep (7).

During the COVID-19 quarantine, a rise in the prevalence of anxiety and depression symptoms has been reported in comparison with the prevalence in general population prior to the pandemic (15). Several systematic reviews and meta-analyses show that the prevalence of anxiety and depressive symptoms varies between 6.33 and 50.9% and 14.6 and 48.3%, respectively, during the peak of the pandemic (16–23). Although previous studies (15, 24) and studies performed during the pandemic (16, 18, 23) have suggested that women may be more affected by symptoms of anxiety and depression than men, this does not correlate with some systematic reviews during the COVID-19 peak (19, 20), and some studies have even found that men have more anxiety symptoms (25). These controversial findings could be due to sociocultural aspects that do not allow extrapolation of these results in LAC countries. Similarly, people of non-binary gender have a high prevalence of disorders related to mental health (26). A multicenter study in countries in Europe and Southeast Asia suggested that mental health disorders increased during the pandemic (27). However, as with results in women, sociocultural aspects do not allow their extrapolation in countries in Latin America.

Although some studies that evaluate mental health, including depression and anxiety, during the pandemic in the Latin American countries such as Argentina (28), Brazil (29), Mexico (30), and Ecuador have been published (31), the effect of gender on the prevalence of depressive and anxiety symptoms during the pandemic in the LAC countries has not been evaluated. Therefore, our objective was to evaluate this association in the LAC countries during the first stage of the COVID-19 pandemic.

## Methods

### Design and Study Area

We carried out a secondary analysis of a database generated by social network Facebook (Facebook, Inc.) and the University of Maryland. This database was constructed based on a virtual survey, to obtain relevant information at the population level within the context of the COVID-19 pandemic. The survey included five modules: sociodemographic characteristics, contact report, general health, mental health, and economic security throughout the pandemic. It was initially conducted on April 23, 2020 and was translated and adapted according to the regions that use Facebook (32). We published a previous study using this database (33), in which the methodology was described in greater detail.

### Population and Sample

The population for this study included users of the Facebook platform over 18 years of age from the LAC countries, which corresponded to 1,440,586 people from 20 countries. People who answered the modules of mental health and sociodemographic characteristics were considered for the analysis, and our exclusion criterion was the absence of complete information related to the variables of interest. The final sample analyzed included 1,338,320 participants. The analysis period comprised surveys from April 23 to May 23, 2020.

### Variables and Procedure

#### Outcomes

##### Depressive Symptomatology

This was evaluated using one adapted question from the Kessler Psychological Distress Scale (K10): “In the past seven days, how often did you feel depressed?” This question allowed five answers (all of the time, most of the time, some of the time, a little of the time, and none of the time) (34). The variable was dichotomized as absence of depressive symptoms when the selected answer was “none of the time” and presence of depressive symptoms in any of the four remaining options.

##### Anxiety Symptomatology

This was evaluated using the K10 adapted question: “During the last seven days, how often did you feel so nervous that nothing could calm you down?” This question allowed five possible answers (all of the time, most of the time, some of the time, a little of the time, and none of the time) (34). This variable was dichotomized considering the absence of anxiety symptoms as none of the time and the presence of anxiety symptoms as any of the four remaining options.

The K10 is a short scale that is easily applied by first-level care personnel and has been used in different studies at the population level. This scale has a Spanish translation and has been applied in various studies in Spain, Colombia, Mexico, and Peru (34, 35), making it an appropriate instrument for the purposes of this research.

#### Independent Variables

Sociodemographic information was included and categorized as gender (categorized as male, female, and non-binary), age (presented as 18–24, 25–34, 35–44, 45–54, 55–64, 65–74, and 75 or more years), and area of residence (town, rural area, and city).

Likewise, suspicious symptoms of COVID-19 were included as a covariate, defined as the presence of three or more symptoms compatible with an acute infection of COVID-19 (cough, fever, loss of smell, fatigue, chest pain, headache, coryza, respiratory distress, eye pain, sore throat, muscle pain, and nausea) in line with the World Health Organization (WHO) definition of a suspected case (36).

In addition, we considered as covariate the compliance with the principal community mitigation strategies (CMS) (physical distancing, washing of hands, and mask using). Compliance with physical distancing was stablished as not having direct contact (even kissing, touching, hugging, and hand shaking) in the last 24 h for more than 1 min and within 2 m of anyone that does not share the same household. Handwashing compliance was defined as a report of at least one handwash after being in public during the last 7 days. Likewise, compliance with the use of a mask was defined as wearing a mask at least once in public within the prior 7 days.

Food insecurity was evaluated with the question: “How worried are you about having enough to eat in the next week?” which allowed the following responses: very worried, somewhat worried, not too worried, and not worried at all. The first three options were defined as a presence of food insecurity.

The fear that the participant or a member of the participant's family could fall seriously ill from COVID-19 was also included as a covariate and was assessed with the following question: “How worried are you that you or someone in your immediate family might become seriously ill from coronavirus (COVID-19)?,” and its four probable answers: very worried, somewhat worried, not too worried, and not worried at all. Then we stablished “not worried at all” as the lack of fear that the participant or any member of the participant's family could be affected by COVID-19 and any of the other three options as the presence of this condition.

#### Statistical Analysis

The database was downloaded in Microsoft Excel sheets and imported into the statistical software Stata v14.0 (StataCorp, TX, USA). All analyses considered the survey's complex sampling via the “svy” command.

Qualitative variables were described using weighted proportions according to complex sampling, with their 95% confidence intervals (95% CI) and absolute frequencies. The bivariate analysis between the outcomes (depression and anxiety symptomatology) and the covariates were performed using the Chi-squared test with Rao–Scott correction taking into account the survey's complex sampling. To explore factors associated with anxiety or depression symptomatology, we employed generalized linear models of the Poisson family with logarithmic link function. Crude and adjusted prevalence ratios (PR) and 95% CIs were estimated. In the adjusted model, we included only the variables with a statistically significant *p*-value (*p* < 0.05) obtained from the crude model. In addition, we evaluated the possible interaction of gender using the adjusted Wald test.

#### Ethical Aspects

Authorization was obtained from the University of Maryland to carry out the study and the institution that provided access to the analyzed database. The database was downloaded without identifiers of the survey participants, safeguarding the non-identification of participants.

## Results

### Characteristics of the Study Population

We analyzed the data of 1,338,320 adults from the LAC countries who answered the survey during April–May 2020. A total of 48.0% (*n* = 590,950) of the participants were men, while 50.6% (*n* = 732,701) and 1.4% (*n* = 14,669) were women and non-binary, respectively. Among the participants, 61.5% (*n* = 1,012,541) were <45 years of age. Suspected symptoms of COVID-19 were described in 18.6% (*n* = 280,168), and 75.8% (*n* = 1,028,162) reported food insecurity. Additionally, 92.3% (*n* = 1,251,992) reported feeling worried about falling seriously ill or that a family member would fall seriously ill from COVID-19. The prevalence of anxiety and depressive symptoms was 44.8% (*n* = 640,418) and 46.6% (*n* = 678,932), respectively (Table 1). The bivariate analysis showed statistically significant differences among anxiety symptoms or depressive symptomatology and the variables of interest (Table 2).

**Table 1:** Descriptive and bivariate analysis of the study sample characteristics (*n* = 1,338,320; *N* = 11,524,713).

	**Total**		
**Characteristics**	**Absolute frequency of participants surveyed**	**Weighted proportion according to each category**	
	***n***	**%**	**95% CI**
**Age (years)**			
18–24	317,015	18.1	17.4–18.8
25–34	412,580	24.8	24.1–25.5
35–44	282,946	18.6	18.3–19.0
45–54	179,402	18.7	18.4–19.0
55–64	104,614	11.1	10.7–11.6
65–74	35,690	7.5	16.9–8.0
75 years or older	6,073	1.2	1.1–1.3
**Living area**			
City	1,052,221	78.9	75.7–81.8
Town	186,023	13.8	11.5–16.5
Village or rural area	100,076	7.3	6.6–8.0
**COVID-19 symptomatology**			
No	1,058,152	81.4	80.5–82.3
Yes	280,168	18.6	17.7–19.5
**Physical distancing**			
No	570,523	40.5	39.1–42.0
Yes	767,797	59.5	58.0–60.9
**Handwashing**			
No	163,022	13.3	12.3–14.4
Yes	1,175,298	86.7	85.6–87.7
**Mask or face covering use**			
No	215,551	17	15.4–18.7
Yes	1,122,769	83	81.3–84.6
**Worried about having enough to eat next week**			
No	310,158	24.2	23.1–25.4
Yes	1,028,162	75.8	74.6–76.9
**Worried about a family member getting sick with COVID-19**			
No	86,328	7.7	7.0–8.5
Yes	1,251,992	92.3	91.5–93.0
**Anxiety symptomatology**			
No	697,902	55.2	54.6–55.8
Yes	640,418	44.8	44.2–45.4
**Depressive symptomatology**			
No	659,388	53.3	52.5–54.0
Yes	678,932	46.7	46.0–47.5

*95% CI, 95% confidence interval*.

**Table 2:** Bivariate analysis of the study sample characteristics according to anxiety or depression (*n* = 1,338,320; *N* = 11,524,713).

	**Anxiety symptomatology**					**Depression symptomatology**				
	**No**		**Yes**			**No**		**Yes**		
**Characteristics**	**Weighted proportion according to each category**		**Weighted proportion according to each category**		***p*-Value**	**Weighted proportion according to each category**		**Weighted proportion according to each category**		***p*-Value**
	**%**	**95% CI**	**%**	**95% CI**		**%**	**95% CI**	**%**	**95% CI**	
**Gender**					<0.001					<0.001
Male	62.0	61.4–62.5	38.0	37.5–38.6		60.6	60.0–61.3	39.4	38.7–40.0	
Female	48.8	47.9–49.6	51.2	50.4–52.1		46.3	45.4–47.2	53.7	52.8–54.6	
Nonbinary	55.3	52.9–57.6	44.7	42.4–47.1		52.7	49.7–55.6	47.3	44.4–50.3	
**Age (years)**					<0.001					<0.001
18–24	47.7	46.5–48.9	52.3	51.1–53.5		38.4	37.6–39.3	61.6	60.7–62.4	
25–34	51.3	50.4–52.3	48.7	47.7–49.6		48.5	47.5–49.5	51.5	50.5–52.5	
35–44	54.5	53.6–55.5	45.5	44.5–46.4		54.6	53.5–55.7	45.4	44.3–46.5	
45–54	58.6	57.9–59.4	41.4	40.6–42.1		59.6	58.6–60.6	40.4	39.4–41.4	
55–64	62.8	61.8–63.8	37.2	36.2–38.2		64.1	63.1–65.2	35.9	34.8–36.9	
65–74	66.1	64.9–67.2	33.9	32.8–35.1		67.1	65.9–68.2	32.9	31.8–34.1	
75 years or older	67.4	64.5–70.2	32.6	29.8–35.5		68.7	66.3–71.0	31.3	29.0–33.7	
**Living area**					<0.001					<0.001
City	54.5	53.9–55.1	45.5	44.9–46.1		52.8	52.1–53.6	47.2	46.4–47.9	
Town	56.5	55.6–57.3	43.5	42.7–44.4		53.3	52.3–54.2	46.7	45.8–47.7	
Village or rural area	60.5	59.4–61.5	39.5	38.5–40.6		57.9	56.7–59.0	42.1	41.0–43.3	
**COVID-19 symptomatology**					<0.001					<0.001
No	60.0	59.4–60.6	40.0	39.4–40.6		58.3	57.5–59.1	41.7	40.9–42.5	
Yes	34.2	33.5–34.9	65.8	65.1–66.5		31.2	30.4–32.0	68.8	68.0–70.0	
**Physical distancing**					<0.001					<0.001
No	53.0	52.4–53.7	47.0	46.3–47.6		50.5	49.8–51.2	49.5	48.8–50.2	
Yes	56.7	56.0–57.4	43.3	42.6–44.0		55.1	54.2–56.0	44.9	44.0–45.8	
**Handwashing**					<0.001					<0.001
No	60.1	59.2–61.0	39.9	39.0–40.8		56.2	55.0–57.3	43.8	42.7–45.0	
Yes	54.5	53.9–55.0	45.5	45.0–46.1		52.8	52.1–53.5	47.2	46.5–47.9	
**Mask or face covering use**					<0.001					<0.001
No	59.6	58.5–60.8	40.4	39.2–41.5		55.6	54.3–56.8	44.4	43.2–45.7	
Yes	54.3	53.8–54.9	45.7	45.1–46.2		52.8	52.1–53.5	47.2	46.5–47.9	
**Food insecurity**					<0.001					<0.001
No	70.4	69.3–71.5	29.6	28.5–30.7		69.4	68.2–70.7	30.6	29.3–31.8	
Yes	50.3	49.7–51.0	49.7	49.0–50.3		48.1	47.3–48.9	51.9	51.1–52.7	
**Worried about becoming seriously ill or a family member become seriously ill with COVID-19**					<0.001					<0.001
No	83.4	82.6–84.3	16.6	15.7–17.4		79.8	78.8–80.7	20.2	19.3–21.2	
Yes	52.8	52.3–53.3	47.2	46.7–47.7		51.0	50.4–51.7	49.0	48.3–49.6	

*95% CI, 95% confidence interval*.

### Prevalence of Anxiety and Depressive Symptoms According to Country

**Figure 1:**
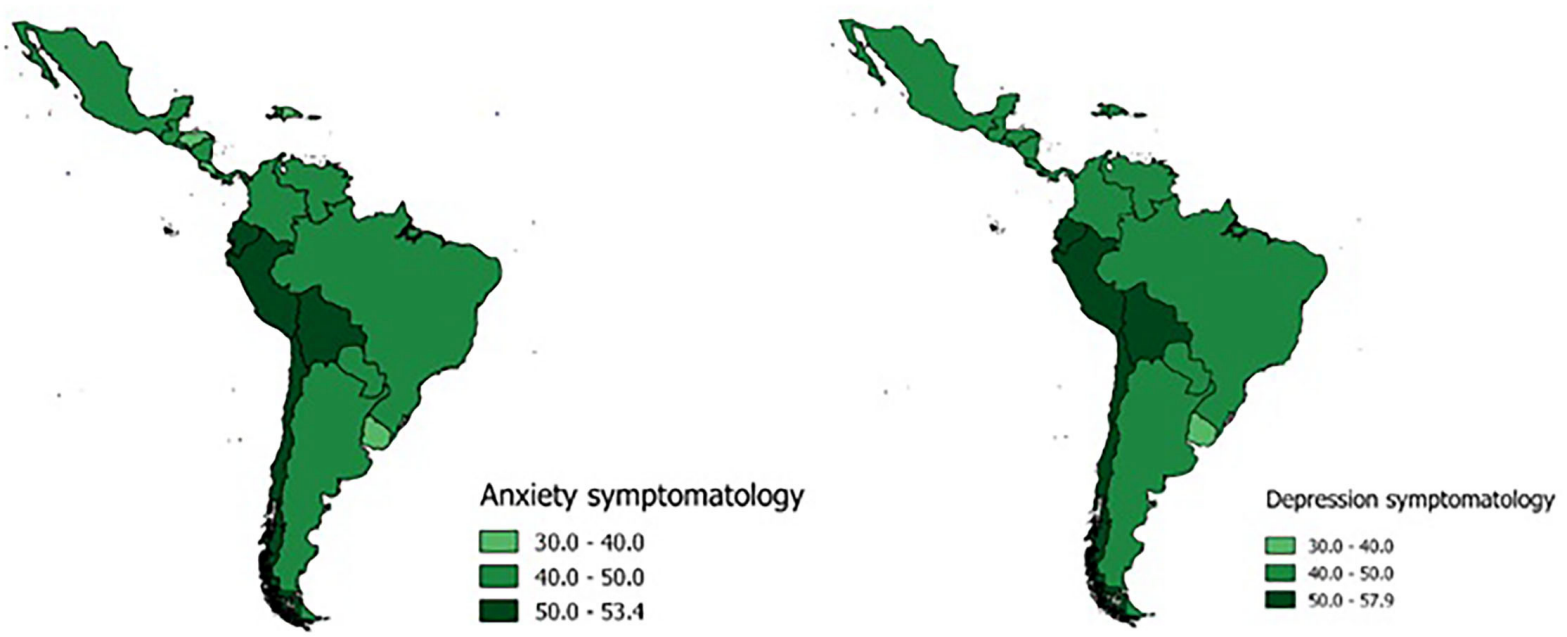
Anxiety and depression prevalences according to the Latin American and the Caribbean countries.

Countries with the highest prevalence of both anxiety and depression were Bolivia (43.2%; 95% CI: 42.5–44.0), Chile (41.3%; 95% CI: 40.2–42.5), Ecuador (41.3%; 95% CI: 40.1–42.6), and Peru (40.1%; 95% CI: 39.1–41.1). Likewise, those with the lowest prevalence of both symptoms were Uruguay (27.1%; 95% CI: 25.5–28.7), Dominican Republic (27.1%; 95% CI: 25.7–28.7), Costa Rica (29.0%; 95% CI: 28.2–29.9), and Honduras (29.5%; 95% CI: 28.3–30.8) (Figure 1).

### Factors Associated With Anxiety Symptomatology

The adjusted model determined that women (PR = 1.30; 95% CI: 1.28–1.31) and non-binary gender (PR = 1.20; 95% CI: 1.15–1.25) had a higher prevalence of anxiety symptomatology compared with males. In addition, being 25–34 years old (PR = 0.94; 95% CI: 0.93–0.95), 35–44 (PR = 0.91; 95% CI: 0.90–0.92), 45–54 (PR = 0.86; 95% CI: 0.84–0.88), 55–64 (PR = 0.83; 95% CI: 0.80–0.86), 65–74 (PR = 0.82; 95% CI: 0.78–0.86), 75 years or older (PR = 0.86; 95% CI: 0.78–0.95), compared with being 18–24 years old, was associated with a lower prevalence of anxiety symptoms. Similarly, living in a town (PR = 0.96; 95% CI: 0.94–0.98) or in a village or rural area (PR = 0.90; 95% CI: 0.88–0.91) was associated with a lower prevalence of anxiety symptoms when compared with living in a city. In addition, the presence of suspected symptoms of COVID-19 (PR = 1.47; 95% CI: 1.46–1.49), compliance with handwashing (PR = 1.03; 95% CI: 1.02–1.05), use of a mask (PR = 1.07; 95% CI: 1.05–1.09), food insecurity (PR = 1.45; 95% CI: 1.41–1.49), and being worried about falling seriously ill or a family member become seriously ill with COVID-19 (PR = 2.30; 95% CI: 2.20–2.41) had a higher prevalence of anxiety symptoms. In contrast, compliance with physical distancing (PR = 0.98; 95% CI: 0.97–0.99) showed an association with a lower prevalence of anxiety symptoms (Table 3).

**Table 3:** Crude and adjusted generalized linear models of Poisson family with logarithmic link to evaluate the factors associated with anxiety or depression in the study sample.

	**Anxiety symptomatology**						**Depression symptomatology**					
**Characteristics**	**Crude**			**Adjusted**			**Crude**			**Adjusted**		
	**PR**	**95% CI**	***p*-Value**	**PR**	**95% CI**	***p*-Value**	**PR**	**95% CI**	***p*-Value**	**PR**	**95% CI**	***p*-Value**
**Gender**												
Male	Reference	-	-	Reference	-	-	Reference	-	-	Reference	-	-
Female	1.35	1.32–1.37	<0.001	1.30	1.28–1.31	<0.001	1.37	1.34–1.39	<0.001	1.32	1.30–1.33	<0.001
Non-binary	1.18	1.12–1.23	<0.001	1.20	1.15–1.25	<0.001	1.20	1.14–1.27	<0.001	1.20	1.15–1.25	<0.001
**Age (years)**												
18–24	Reference	-	-	Reference	-	-	Reference	-	-	Reference	-	-
25–34	0.93	0.92–0.94	<0.001	0.94	0.93–0.95	<0.001	0.84	0.83–0.84	<0.001	0.85	0.84–0.86	<0.001
35–44	0.87	0.86–0.88	<0.001	0.91	0.90–0.92	<0.001	0.74	0.73–0.75	<0.001	0.78	0.77–0.79	<0.001
45–54	0.79	0.77–0.81	<0.001	0.86	0.84–0.88	<0.001	0.66	0.65–0.67	<0.001	0.71	0.70–0.72	<0.001
55–64	0.71	0.69–0.74	<0.001	0.83	0.80–0.86	<0.001	0.58	0.57–0.60	<0.001	0.68	0.66–0.69	<0.001
65–74	0.65	0.62–0.68	<0.001	0.82	0.78–0.86	<0.001	0.53	0.52–0.55	<0.001	0.67	0.64–0.69	<0.001
75 years or older	0.62	0.56–0.69	<0.001	0.86	0.78–0.95	0.003	0.51	0.47–0.55	<0.001	0.69	0.64–0.74	<0.001
**Living area**												
City	Reference	-	-	Reference	-	-	Reference	-	-	Reference	-	-
Town	0.96	0.94–0.97	<0.001	0.96	0.94–0.98	<0.001	0.99	0.98–1.00	0.178	0.98	0.96–0.99	0.003
Village or rural area	0.87	0.85–0.89	<0.001	0.90	0.88–0.91	<0.001	0.89	0.87–0.91	<0.001	0.90	0.88–0.92	<0.001
**COVID-19 symptomatology**												
No	Reference	-	-	Reference	-	-	Reference	-	-	Reference	-	-
Yes	1.64	1.63–1.66	<0.001	1.47	1.46–1.49	<0.001	1.65	1.63–1.67	<0.001	1.44	1.43–1.45	<0.001
**Physical distancing**												
No	Reference	-	-	Reference	-	-	Reference	-	-	Reference	-	-
Yes	0.92	0.91–0.94	<0.001	0.98	0.97–0.99	0.004	0.91	0.89–0.92	<0.001	0.97	0.96–0.98	<0.001
**Handwashing**												
No	Reference	-	-	Reference	-	-	Reference	-	-	Reference	-	-
Yes	1.14	1.12–1.16	<0.001	1.03	1.02–1.05	<0.001	1.08	1.06–1.10	<0.001	1.00	0.99–1.02	0.701
**Mask or face covering use**												
No	Reference	-	-	Reference	-	-	Reference	-	-	Reference	-	-
Yes	1.13	1.11–1.16	<0.001	1.07	1.05–1.09	<0.001	1.06	1.04–1.09	<0.001	1.03	1.01–1.05	<0.001
**Food insecurity**												
No	Reference	-	-	Reference	-	-	Reference	-	-	Reference	-	-
Yes	1.68	1.63–1.73	<0.001	1.45	1.41–1.49	<0.001	1.68	1.63–1.73	<0.001	1.44	1.40–1.49	<0.001
**Worried about becoming seriously ill or a family member become seriously ill with COVID-19**												
No	Reference	-	-	Reference	-	-	Reference	-	-	Reference	-	-
Yes	2.85	2.71–2.99	<0.001	2.30	2.20–2.41	<0.001	2.85	2.71–2.99	<0.001	1.94	1.87–2.02	<0.001

*95% CI, 95% confidence interval*.

### Factors Associated With Depressive Symptomatology

In the adjusted model, women participants (PR = 1.32; 95% CI: 1.30–1.33) and non-binary (PR = 1.20; 95% CI: 1.15–1.25) had a higher prevalence of depressive symptomatology in comparison with males. Alternatively, being 25–34 years old (PR = 0.85; 95% CI: 0.84–0.86), 35–44 (PR = 0.78; 95% CI: 0.77–0.79), 45–54 (PR = 0.71; 95% CI: 0.70–0.72), 55–64 (PR = 0.68; 95% CI: 0.66–0.69), 65–74 (PR = 0.67; 95% CI: 0.64–0.69), 75 years or older (PR = 0.69; 95% CI: 0.64–0.74), compared with being 18–24 years old, was associated with a lower prevalence of this symptomatology. Living in a village or rural area (PR = 0.90; 95% CI: 0.88–0.92) and in a town (PR = 0.98; 95% CI: 0.96–0.99), compared with living in a city, was also associated with a lower prevalence. Meanwhile, having suspected symptoms of COVID-19 (PR = 1.44; 95% CI: 1.43–1.45), use of a mask (PR = 1.03; 95% CI: 1.01–1.05), food insecurity (PR = 1.44; 95% CI: 1.40–1.49), and being worried about becoming seriously ill or a family member become seriously ill with COVID-19 (PR = 1.94; 95% CI: 1.87–2.02) were associated with a higher prevalence of depressive symptomatology. By contrast, compliance with physical distancing (PR = 0.97; 95% CI: 0.96–0.98) was associated with a lower prevalence of depressive symptoms (Table 3).

### Factors Associated With Anxiety or Depressive Symptomatology Stratified by Gender

We found interactions between gender and the rest of the independent variables. Compared with the group of men and women, among non-binary participants, after 55 years, the association between age and anxiety symptomatology was lost. Similarly, whereas living in a town was associated with a lower prevalence of anxiety and depression symptomatology in men and women, this did not happen among non-binary participants (anxiety: PRa = 0.97; 95% CI = 0.90–1.05; *p* = 0.445, depression: PRa = 0.95; 95% CI = 0.88–1.03; *p* = 0.221) (Tables 4–6).

**Table 4:** Crude and adjusted generalized linear models of Poisson family with logarithmic link to evaluate the factors associated with anxiety or depression in men participants.

	**Anxiety symptomatology**						**Depression symptomatology**					
**Characteristics**	**Crude**			**Adjusted**			**Crude**			**Adjusted**		
	**PR**	**95% CI**	***p*-Value**	**PR**	**95% CI**	***p*-Value**	**PR**	**95% CI**	***p*-Value**	**PR**	**95% CI**	***p*-Value**
**Age (years)**												
18–24	Reference	-	-	Reference	-	-	Reference	-	-	Reference	-	-
25–34	0.90	0.89–0.91	<0.001	0.92	0.91–0.93	<0.001	0.79	0.8–0.80	<0.001	0.81	0.80–0.82	<0.001
35–44	0.82	0.81–0.83	<0.001	0.87	0.86–0.88	<0.001	0.67	0.66–0.69	<0.001	0.72	0.70–0.73	<0.001
45–54	0.73	0.70–0.75	<0.001	0.80	0.78–0.83	<0.001	0.59	0.57–0.60	<0.001	0.65	0.63–0.66	<0.001
55–64	0.67	0.64–0.69	<0.001	0.78	0.75–0.81	<0.001	0.53	0.52–0.55	<0.001	0.62	0.60–0.64	<0.001
65–74	0.60	0.57–0.64	<0.001	0.77	0.73–0.82	<0.001	0.47	0.45–0.50	<0.001	0.60	0.57–0.63	<0.001
75 years or older	0.61	0.55–0.69	<0.001	0.85	0.76–0.95	0.006	0.48	0.44–0.52	<0.001	0.64	0.59–0.70	<0.001
**Living area**												
City	Reference	-	-	Reference	-	-	Reference	-	-	Reference	-	-
Town	0.99	0.97–1.00	0.070	0.98	0.96–0.99	0.036	1.01	0.99–1.03	0.289	0.98	0.96–1.00	0.058
Village or rural area	0.88	0.85–0.91	<0.001	0.91	0.88–0.94	<0.001	0.88	0.85–0.91	<0.001	0.89	0.86–0.92	<0.001
**COVID-19 symptomatology**												
No	Reference	-	-	Reference	-	-	Reference	-	-	Reference	-	-
Yes	1.77	1.75–1.80	<0.001	1.61	1.59–1.63	<0.001	1.76	1.74–1.79	<0.001	1.55	1.53–1.57	<0.001
**Physical distancing**												
No	Reference	-	-	Reference	-	-	Reference	-	-	Reference	-	-
Yes	0.90	0.88–0.92	<0.001	0.99	0.96–1.01	0.199	0.89	0.88–0.91	<0.001	0.98	0.96–1.00	0.084
**Handwashing**												
No	Reference	-	-	Reference	-	-	Reference	-	-	Reference	-	-
Yes	1.22	1.19–1.25	<0.001	1.06	1.03–1.08	<0.001	1.11	1.08–1.15	<0.001	1.00	0.98–1.03	0.756
**Mask or face covering use**												
No	Reference	-	-	Reference	-	-	Reference	-	-	Reference	-	-
Yes	1.18	1.13–1.23	<0.001	1.08	1.05–1.11	<0.001	1.08	1.04–1.12	<0.001	1.04	1.01–1.06	0.005
**Food insecurity**												
No	Reference	-	-	Reference	-	-	Reference	-	-	Reference	-	-
Yes	1.79	1.73–1.86	<0.001	1.53	1.49–1.58	<0.001	1.80	1.73–1.87	<0.001	1.52	1.47–1.58	<0.001
**Worried about becoming seriously ill or a family member become seriously ill with COVID-19**												
No	Reference	-	-	Reference	-	-	Reference	-	-	Reference	-	-
Yes	2.72	2.59–2.85	<0.001	2.21	2.11–2.31	<0.001	2.31	2.22–2.41	<0.001	1.88	1.81–1.94	<0.001

*95% CI, 95% confidence interval*.

**Table 5:** Crude and adjusted generalized linear models of Poisson family with logarithmic link to evaluate the factors associated with anxiety or depression in women participants.

	**Anxiety symptomatology**						**Depression symptomatology**					
**Characteristics**	**Crude**			**Adjusted**			**Crude**			**Adjusted**		
	**PR**	**95% CI**	***p*-Value**	**PR**	**95% CI**	***p*-Value**	**PR**	**95% CI**	***p*-Value**	**PR**	**95% CI**	***p*-Value**
**Age (years)**												
18–24	Reference	-	-	Reference	-	-	Reference	-	-	Reference	-	-
25–34	0.94	0.93–0.96	<0.001	0.96	0.94–0.97	<0.001	0.86	0.85–0.87	<0.001	0.88	0.87–0.89	<0.001
35–44	0.91	0.90–0.92	<0.001	0.95	0.93–0.96	<0.001	0.79	0.78–0.80	<0.001	0.83	0.82–0.84	<0.001
45–54	0.82	0.81–0.84	<0.001	0.90	0.88–0.92	<0.001	0.70	0.69–0.71	<0.001	0.76	0.75–0.78	<0.001
55–64	0.74	0.71–0.78	<0.001	0.87	0.83–0.91	<0.001	0.62	0.61–0.64	<0.001	0.73	0.71–0.74	<0.001
65–74	0.67	0.64–0.70	<0.001	0.85	0.81–0.90	<0.001	0.57	0.55–0.59	<0.001	0.72	0.70–0.75	<0.001
75 years or older	0.63	0.57–0.70	<0.001	0.85	0.77–0.94	0.002	0.53	0.48–0.58	<0.001	0.70	0.64–0.77	<0.001
**Living area**												
City	Reference	-	-	Reference	-	-	Reference	-	-	Reference	-	-
Town	0.94	0.92–0.96	<0.001	0.94	0.92–0.97	<0.001	0.99	0.97–1.00	0.148	0.97	0.96–0.99	0.002
Village or rural area	0.88	0.86–0.89	<0.001	0.89	0.88–0.91	<0.001	0.91	0.89–0.93	<0.001	0.91	0.90–0.93	<0.001
**COVID-19 symptomatology**												
No	Reference	-	-	Reference	-	-	Reference	-	-	Reference	-	-
Yes	1.51	1.49–1.52	<0.001	1.40	1.38–1.41	<0.001	1.52	1.50–1.53	<0.001	1.38	1.36–1.39	<0.001
**Physical distancing**												
No	Reference	-	-	Reference	-	-	Reference	-	-	Reference	-	-
Yes	0.90	0.89–0.92	<0.001	0.98	0.97–0.99	<0.001	0.88	0.87–0.89	<0.001	0.96	0.95–0.97	<0.001
**Handwashing**												
No	Reference	-	-	Reference	-	-	Reference	-	-	Reference	-	-
Yes	1.17	1.15–1.19	<0.001	1.03	1.01–1.05	0.016	1.13	1.11–1.14	<0.001	1.01	0.99–1.02	0.404
**Mask or face covering use**												
No	Reference	-	-	Reference	-	-	Reference	-	-	Reference	-	-
Yes	1.16	1.14–1.18	<0.001	1.06	1.04–1.08	<0.001	1.11	1.08–1.13	<0.001	1.03	1.01–1.05	0.010
**Food insecurity**												
No	Reference	-	-	Reference	-	-	Reference	-	-	Reference	-	-
Yes	1.58	1.54–1.62	<0.001	1.39	1.35–1.43	<0.001	1.61	1.56–1.66	<0.001	1.39	1.35–1.44	<0.001
**Worried about becoming seriously ill or a family member become seriously ill with COVID-19**												
No	Reference	-	-	Reference	-	-	Reference	-	-	Reference	-	-
Yes	2.84	2.67–3.02	<0.001	2.38	2.25–2.52	<0.001	2.39	2.26–2.53	<0.001	1.98	1.88–2.09	<0.001

*95% CI, 95% confidence interval*.

**Table 6:** Crude and adjusted generalized linear models of Poisson family with logarithmic link to evaluate the factors associated with anxiety or depression in non-binary participants.

	**Anxiety symptomatology**						**Depression symptomatology**					
**Characteristics**	**Crude**			**Adjusted**			**Crude**			**Adjusted**		
	**PR**	**95% CI**	***p*-Value**	**PR**	**95% CI**	***p*-Value**	**PR**	**95% CI**	***p*-Value**	**PR**	**95% CI**	***p*-Value**
**Age (years)**												
18–24	Reference	-	-	Reference	-	-	Reference	-	-	Reference	-	-
25–34	0.90	0.84–0.97	0.005	0.92	0.85–0.99	0.039	0.75	0.69–0.80	<0.001	0.77	0.71–0.83	<0.001
35–44	0.85	0.79–0.92	<0.001	0.92	0.85–0.98	0.016	0.68	0.62–0.74	<0.001	0.74	0.69–0.79	<0.001
45–54	0.74	0.65–0.83	<0.001	0.84	0.76–0.92	<0.001	0.61	0.55–0.66	<0.001	0.69	0.64–0.75	<0.001
55–64	0.79	0.67–0.92	0.002	0.91	0.81–1.02	0.108	0.63	0.52–0.76	<0.001	0.74	0.64–0.84	<0.001
65–74	0.75	0.65–0.87	<0.001	0.96	0.83–1.12	0.631	0.53	0.43–0.66	<0.001	0.69	0.56–0.84	<0.001
75 years or older	0.68	0.27–1.70	0.413	1.14	0.56–2.32	0.726	0.67	0.33–1.35	0.264	1.11	0.67–1.84	0.680
**Living area**												
City	Reference	-	-	Reference	-	-	Reference	-	-	Reference	-	-
Town	0.93	0.85–1.01	0.087	0.97	0.90–1.05	0.445	0.91	0.84–0.98	0.019	0.95	0.88–1.03	0.221
Village or rural area	0.81	0.72–0.90	<0.001	0.88	0.79–0.97	0.009	0.87	0.79–0.97	0.009	0.94	0.86–1.02	0.116
**COVID-19 symptomatology**												
No	Reference	-	-	Reference	-	-	Reference	-	-	Reference	-	-
Yes	1.68	1.54–1.83	<0.001	1.51	1.41–1.62	<0.001	1.80	1.64–1.97	<0.001	1.57	1.47–1.68	<0.001
**Physical distancing**												
No	Reference	-	-	Reference	-	-	Reference	-	-	Reference	-	-
Yes	0.84	0.78–0.90	<0.001	0.92	0.88–0.98	0.005	0.85	0.76–0.95	0.005	0.95	0.89–1.02	0.200
**Handwashing**												
No	Reference	-	-	Reference	-	-	Reference	-	-	Reference	-	-
Yes	1.20	1.11–1.30	<0.001	0.99	0.91–1.09	0.855	1.16	1.08–1.24	<0.001	0.99	0.93–1.06	0.877
**Mask or face covering use**												
No	Reference	-	-	Reference	-	-	Reference	-	-	Reference	-	-
Yes	1.25	1.17–1.34	<0.001	1.16	1.06–1.28	0.002	1.19	1.11–1.29	<0.001	1.13	1.02–1.26	0.015
**Food insecurity**												
No	Reference	-	-	Reference	-	-	Reference	-	-	Reference	-	-
Yes	1.77	1.49–2.09	<0.001	1.44	1.27–1.63	<0.001	1.78	1.54–2.05	<0.001	1.44	1.31–1.58	<0.001
**Worried about becoming seriously ill or a family member become seriously ill with COVID-19**												
No	Reference	-	-	Reference	-	-	Reference	-	-	Reference	-	-
Yes	2.98	2.43–3.66	<0.001	2.45	1.93–3.12	<0.001	2.75	2.28–3.33	<0.001	2.26	1.86–2.75	<0.001

*95% CI, 95% confidence interval*.

Women who complied with physical distancing had a lower prevalence of anxiety and depression symptomatology (anxiety: PRa = 0.98; 95% CI = 0.97–0.99; *p* < 0.001, depression: PRa = 0.96; 95% CI = 0.95–0.97; *p* < 0.001), while this was observed only in the case of anxiety for nonbinary participants (anxiety: PRa = 0.92; 95% CI = 0.88–0.98; *p* = 0.005). Men participants did not show lower prevalence for neither symptomatology (anxiety: PRa = 0.99; 95% CI = 0.96–1.01; *p* = 0.199, depression: PRa = 0.98; 95% CI = 0.96–1.00; *p* = 0.084). Yet, we found association between compliance with handwashing and a higher prevalence of anxiety symptomatology among men (PRa = 1.06; 95% CI = 1.05–1.11; *p* < 0.001) and women participants (PRa = 1.03; 95% CI = 1.01–1.05; *p* = 0.016), but not among non-binary participants (PRa = 0.99; 95% CI = 0.91–1.09; *p* = 0.855) (Tables 4–6).

## Discussion

### Main Findings

The study's goal was to evaluate factors associated with depressive and anxiety symptoms in the LAC countries during the first stage of quarantine due to COVID-19. In addition, we explored the gender differences on the presence of depressive or anxiety symptoms. We found that almost half of the participants had symptoms of anxiety or depression at the beginning of the pandemic. Four countries in the South American region presented the highest prevalence of anxiety or depression symptoms compared with other countries.

### Previous Studies

The studies published in some LAC countries showed results that differ from ours, but they also differ in the populations evaluated and the instruments used. A Mexican study carried out in the general population that used the PHQ-9, the GAD-7, and a visual analog scale, found that 20.8% had severe anxiety symptoms, and 27.5% had severe depressive symptoms (30). In Argentina, the Brief-Symptom Inventory-53 was used to evaluate various aspects of mental health, and it was found that, in the general population evaluated, 31.8% presented symptoms of anxiety and 27.5% presented symptoms of depression (28). Finally, a Brazilian study that elaborated its own questionnaire found that 40.4% frequently had feelings of sadness or depression, and 52.6% were frequently nervous or anxious (29). We only found one previous study that evaluated psychological distress using the K10, but it was carried out in college students from Argentina, and then it would not be comparable with our study (37). Our research evaluated the general population, and therefore, it is only comparable with studies from Argentina (28) and Brazil (29).

Previous studies carried out in Spain (38–40), Turkey (41), and China (42) intended to assess factors associated with depressive and anxiety symptomatology with a gender-based approach. However, only one study carried out in Spanish adults evaluated the factors associated with anxiety and depression, stratifying by gender; however, they did not consider the non-binary gender, and measured anxiety and depression with the GAD-7 and PHQ-9, respectively. The rest of the studies considered did not include non-binary gender in their analysis; so, our study will contribute by characterizing this population. In addition, we did not find previous studies with this objective carried out in adults from the LAC countries.

### Interpretation of the Results

Although various meta-analyses and systematic reviews have been published in relation to the prevalence of depression and anxiety symptoms during the COVID-19 pandemic, the results among the different studies differ (16–23). These differences could be due to variations in the measurement instruments, cutoffs used to define mild to severe symptoms, and the eligibility criteria (16–23). However, mental health was affected differently according to regions and countries in the LAC countries during the pandemic's first wave (43). Likewise, other factors that influence the results by country included differences in the economies of the nations, government preparation to respond to the crisis, the availability of supplies or medical facilities, as well as cultural differences (16). Similarly, adequate dissemination of information related to COVID-19 in each region also influenced the psychological responses of the population, especially during the introduction of quarantines, when lifestyle was jeopardized by mandatory isolation and unexpected unemployment (14, 17).

Among men participants, compliance of physical distancing had association with a lower prevalence of anxiety or depression symptomatology. This could be related to the fact that men usually have less compliance rates of physical distancing (44). Additionally, during the pandemic, they probably had to continue working, making it even more difficult to achieve this compliance, whereas we found a higher prevalence of both anxiety and depression symptomatology among women participants, with results similar to those found in previous systematic reviews and meta-analyses (16, 18, 23). This could be related to a lack of emotional support during the quarantine or differential neurobiological responses when exposed to stressors (45, 46). Likewise, despite improvements in gender roles, historically, women had limited access to education and are more oriented to household tasks. So, several cases of intimate partner violence during this period have been reported worldwide (47). Another possible explanation could lie in the lockdown effect in the work environment, housework, and childcare. Due to the traditional gender division of labor, most of the increased burden of housework and childcare at home during the COVID-19 pandemic were felt on women (48, 49).

The sexual minority population is exposed to a variety of social stressors, such as discrimination, prejudice, and stigma that contribute to increased mental health problems (26, 27). During the pandemic, a multinational study found that women and non-binary gender people reported a higher prevalence of symptoms in every measure of distress (50). In another multinational study of 76 countries, in which 68.8% of the participant's gender was non-binary, it was found that approximately half of those evaluated had depression and anxiety (27). Then the prevalence of anxiety, depressive symptoms, and suicidal ideation increased during the pandemic (27). We did not find an association between age and anxiety symptoms among non-binary participants after 55 years of age, in contrast to men and women. Although the usual onset for anxiety and depression is young adulthood (51), we consider that our results may also be due to a lack of identification with the non-binary gender in older participants (52). Similarly, we found that in unlike men and women participants, among non-binary participants, living in a town was not significantly associated with a lower prevalence of depression and anxiety symptoms. This could be related to the fact that non-binary individuals who live in town and rural areas are usually more likely to restrain their gender expressions (53).

Among the countries evaluated, we found different prevalences of anxiety and depression symptoms; however, there was a higher prevalence of both parameters in Bolivia. Information on mental health and the impact of mental disorders in Bolivia is scarce. According to statistics reported by admissions from psychiatric hospitals around the country, the leading reason for hospitalization is substance abuse, with alcoholism being responsible for 90%, along with being one of the main causes of deaths in traffic accidents and an important risk factor for domestic violence (54). In 2014, 0.46 psychologist and 1.06 psychiatrists were reported per 100,000 inhabitants. Although Bolivia has a National Mental Health Plan that guides the promotion, prevention, treatment, and rehabilitation of mental health from mental illnesses, resources are limited (54). Hence, there is a need for a mental health and gender approach to promote CMS during the pandemic in Bolivia. This approach will be useful also in other Latin American and Caribbean countries.

### Limitations and Strengths

Our study has limitations. Despite being a multinational study with a significant sample, it is based on the users of a social network that not everyone can access. However, it is a widely used social network in Latin America (four out of five Latin American Internet users have a profile on Facebook). Second, the variables included, and their definition, are subject to the pre-established definition of the matrix survey, thus, we could not include culture-related variables. Third, the data were obtained by self-reporting, and therefore, information may be underreported. Fourth, causalities cannot be established between the variables evaluated due to the study design. Fifth, there may be an underreporting of non-binary gender people. Sixth, the questions used are part of a validated instrument; however, they do not constitute one. Finally, the instrument used has not been used in other studies during the pandemic, thereby limiting comparison of the results with other studies. Nonetheless, this is the first multinational study with a significant sample size carried out in the LAC countries.

## Conclusions

In conclusion, nearly 4 out of 10 people had symptoms of anxiety, while almost 5 out of 10 had symptoms of depression. Women and non-binary gender people had more symptoms of anxiety and depression. It is necessary to evaluate strategies according to gender to reduce the impact on mental health in the COVID-19 pandemic. The factors associated with symptoms of anxiety or depression varied according to gender.

## Data Availability Statement

The datasets presented in this article are not readily available because it is a secondary dataset provided by the Maryland University that cannot be publicly shared due to a signed agreement. Requests to access the datasets should be directed to vbeniteszapata@gmail.com.

## Ethics Statement

Ethical review and approval was not required for the study on human participants in accordance with the local legislation and institutional requirements. The patients/participants provided their written informed consent to participate in this study.

## Author Contributions

PH-A, DU-P, VB-Z, GB-Q, CT-H, and AH participated in the conception of the article, the data collection, the writing of the article, and the approval of the final version. DU-P and VB-Z performed the data analysis. All authors contributed to the article and approved the submitted version.

## Conflict of Interest

The authors declare that the research was conducted in the absence of any commercial or financial relationships that could be construed as a potential conflict of interest.

## Publisher's Note

All claims expressed in this article are solely those of the authors and do not necessarily represent those of their affiliated organizations, or those of the publisher, the editors and the reviewers. Any product that may be evaluated in this article, or claim that may be made by its manufacturer, is not guaranteed or endorsed by the publisher.
