# Supplementary material for: Gender Differences in Depressive and Anxiety Symptoms During the First Stage of the COVID-19 Pandemic: A Cross-Sectional Study in Latin America and the Caribbean
Source: Front Psychiatry. 2022 Mar 17;13:727034. doi: 10.3389/fpsyt.2022.727034 (PMC8968114; doi:10.3389/fpsyt.2022.727034)
Supplement: Supplementary file 1 [file Data_Sheet_1.docx]

**Supplementary material**

| Table 7. Descriptive and bivariate analysis of the study sample characteristics according to gender (Male+Female vs. non-binary) (n=1,338,320; N=11,524,713). | | | | | |
| --- | --- | --- | --- | --- | --- |
|  | **Gender** | | | | |
|  | Male+Female | | Non-binary | |  |
| Characteristics | Weighted proportion according to each category | | Weighted proportion according to each category | | p value |
|  | % | 95%CI | % | 95%CI |  |
| Age (years) |  |  |  |  | <0.001 |
| 18-24 | 18.0 | 17.3-18.7 | 23.0 | 20.1-26.2 |  |
| 25-34 | 24.8 | 24.1-25.5 | 23.4 | 21.6-25.3 |  |
| 35-44 | 18.7 | 18.4-19.0 | 16.5 | 15.0-18.1 |  |
| 45-54 | 18.7 | 18.4-19.1 | 17.2 | 15.7-18.7 |  |
| 55-64 | 11.1 | 10.7-11.6 | 10.4 | 9.1-12.0 |  |
| 65-74 | 7.5 | 6.9-8.1 | 6.8 | 5.7-8.2 |  |
| 75 years or older | 1.2 | 1.1-1.3 | 2.7 | 1.6-4.5 |  |
| Living area |  |  |  |  | <0.001 |
| City | 79.1 | 75.9-81.9 | 68.6 | 64.0-72.9 |  |
| Town | 13.7 | 11.4-16.4 | 20.8 | 17.3-24.8 |  |
| Village or rural area | 7.2 | 6.5-8.0 | 10.6 | 9.2-12.2 |  |
| COVID-19 symptomatology |  |  |  |  | 0.908 |
| No | 81.4 | 80.5-82.3 | 81.5 | 79.8-83.2 |  |
| Yes | 18.6 | 17.7-19.5 | 18.5 | 16.8-20.2 |  |
| Physical distancing |  |  |  |  | <0.001 |
| No | 40.6 | 39.3-42.1 | 33.7 | 30.3-37.3 |  |
| Yes | 59.4 | 57.9-60.7 | 66.3 | 62.7-69.7 |  |
| Hand washing |  |  |  |  | <0.001 |
| No | 13.2 | 12.2-14.3 | 21.2 | 19.0-23.6 |  |
| Yes | 86.8 | 85.7-87.8 | 78.8 | 76.4-81.0 |  |
| Mask or face covering use |  |  |  |  | <0.001 |
| No | 16.8 | 15.3-18.5 | 26.7 | 23.4-30.2 |  |
| Yes | 83.2 | 81.5-84.7 | 73.3 | 69.8-76.6 |  |
| Food insecurity |  |  |  |  | 0.285 |
| No | 24.3 | 23.1-25.4 | 22.9 | 20.0-26.0 |  |
| Yes | 75.7 | 74.6-76.9 | 77.1 | 74.0-80.0 |  |
| Worried about becoming seriously ill or a family member become seriously ill with COVID-19 |  |  |  |  | <0.001 |
| No | 7.6 | 7.0-8.4 | 14.2 | 12.7-15.8 |  |
| Yes | 92.4 | 91.6-93.0 | 85.8 | 84.2-87.3 |  |
| Anxiety symptomatology |  |  |  |  | 0.967 |
| No | 55.2 | 54.6-55.8 | 55.3 | 52.9-57.6 |  |
| Yes | 44.8 | 44.2-45.4 | 44.7 | 42.4-47.1 |  |
| Depressive symptomatology |  |  |  |  | 0.640 |
| No | 53.3 | 52.6-54.0 | 52.7 | 49.7-55.6 |  |
| Yes | 46.7 | 46.0-47.4 | 47.3 | 44.4-50.3 |  |

95%CI. 95% confidence intervals.

| Table 8. Proportion of anxiety or depression according to countries included in the study sample. | | | | | | | | | | | | | | |  |
| --- | --- | --- | --- | --- | --- | --- | --- | --- | --- | --- | --- | --- | --- | --- | --- |
|  | Total | | | **Anxiety symptomatology** | | | | | **Depressive symptomatology** | | | | | |  |
|  | Absolute frequency of participants surveyed | Weighted proportion according to each category | | No | | Yes | | | No | | | Yes | | |  |
| Countries | n | % | 95%CI | % | 95%CI | | % | 95%CI | | % | 95%CI | | % | 95%CI | |
| Argentina | 121,926 | 8.2 | 3.9-16.2 | 54 | 53.2-54.9 | | 46 | 45.1-46.8 | | 52.8 | 51.7-53.8 | | 47.2 | 46.2-48.3 | |
| Bolivia | 27,329 | 1.4 | 0.6-3.1 | 46.6 | 45.7-47.5 | | 53.4 | 52.5-54.3 | | 42.1 | 40.8-43.4 | | 57.9 | 56.6-59.2 | |
| Brazil | 393,779 | 37.1 | 23.9-52.5 | 54.9 | 54.1-55.7 | | 45.1 | 44.3-45.9 | | 53.1 | 52.2-54.0 | | 46.9 | 46.0-47.8 | |
| Chile | 56,572 | 3.4 | 1.6-6.8 | 48.7 | 47.5-49.9 | | 51.3 | 50.1-52.5 | | 46.5 | 45.2-47.8 | | 53.5 | 52.2-54.8 | |
| Colombia | 97,923 | 8.1 | 4.4-14.4 | 58.1 | 57.2-59.1 | | 41.9 | 40.9-42.8 | | 53.6 | 52.6-54.5 | | 46.4 | 45.5-47.4 | |
| Costa Rica | 27,625 | 0.9 | 0.4-1.8 | 61.3 | 60.4-62.3 | | 38.7 | 37.7-39.6 | | 58.3 | 57.2-59.4 | | 41.7 | 40.6-42.8 | |
| Dominican Republic | 17,504 | 1.5 | 0.8-2.9 | 64.5 | 62.9-66.1 | | 35.5 | 33.9-37.1 | | 58.7 | 57.6-59.8 | | 41.3 | 40.2-42.4 | |
| Ecuador | 46,480 | 2.6 | 1.3-5 | 48.8 | 47.6-50.0 | | 51.2 | 50.0-52.4 | | 45.6 | 44.4-46.8 | | 54.4 | 53.2-55.6 | |
| El Salvador | 27,194 | 1 | 0.5-2.1 | 54.4 | 52.8-55.9 | | 45.6 | 44.1-47.2 | | 52.7 | 51.1-54.2 | | 47.3 | 45.8-48.9 | |
| Guatemala | 31,740 | 2.4 | 1.3-4.4 | 58 | 56.5-59.6 | | 42 | 40.4-43.5 | | 54.5 | 53.5-55.6 | | 45.5 | 44.4-46.5 | |
| Haiti | 746 | 0.5 | 0.1-1.5 | 58.4 | 51.6-65.0 | | 41.6 | 35.0-48.4 | | 54.2 | 49.6-58.8 | | 45.8 | 41.2-50.4 | |
| Honduras | 21,117 | 1.4 | 0.7-2.6 | 61.4 | 59.8-63.0 | | 38.6 | 37.0-40.2 | | 56.1 | 55.0-57.1 | | 43.9 | 42.9-45.0 | |
| Mexico | 297,767 | 19.2 | 11.1-31.1 | 57.7 | 56.8-58.6 | | 42.3 | 41.4-43.2 | | 57.8 | 57.1-58.6 | | 42.2 | 41.4-42.9 | |
| Nicaragua | 24,111 | 0.9 | 0.4-2 | 50.7 | 47.7-53.6 | | 49.3 | 46.4-52.3 | | 50 | 47.8-52.1 | | 50 | 47.9-52.2 | |
| Panama | 8,855 | 0.6 | 0.3-1.4 | 56.4 | 53.2-59.5 | | 43.6 | 40.5-46.8 | | 53.7 | 51.9-55.5 | | 46.3 | 44.5-48.1 | |
| Paraguay | 10,856 | 0.8 | 0.4-1.7 | 50.4 | 48.9-52.0 | | 49.6 | 48.0-51.1 | | 53.2 | 51.7-54.7 | | 46.8 | 45.3-48.3 | |
| Peru | 58,631 | 4.8 | 2.4-9.3 | 49.1 | 48.4-49.9 | | 50.9 | 50.1-51.6 | | 46.8 | 45.6-48.1 | | 53.2 | 51.9-54.4 | |
| Puerto Rico, U.S. | 29,730 | 0.5 | 0.3-0.9 | 52.8 | 51.0-54.6 | | 47.2 | 45.4-49.0 | | 52.5 | 50.2-54.8 | | 47.5 | 45.2-49.8 | |
| Uruguay | 20,127 | 0.6 | 0.3-1.2 | 61.5 | 59.4-63.4 | | 38.5 | 36.6-40.6 | | 60.8 | 59.1-62.5 | | 39.2 | 37.5-40.9 | |
| Venezuela | 18,308 | 4.3 | 2.2-8 | 55.3 | 54.2-56.3 | | 44.7 | 43.7-45.8 | | 50.6 | 49.7-51.5 | | 49.4 | 48.5-50.3 | |

95%CI: 95% confidence interval.
